# Diagnoses and prescription patterns among users of medications for obstructive airway diseases in Finland

**DOI:** 10.1186/s12890-024-02870-9

**Published:** 2024-01-31

**Authors:** Pekka Juntunen, Petri Salmela, Johanna Pakkasela, Jussi Karjalainen, Lauri Lehtimäki

**Affiliations:** 1 Department of Respiratory Medicine, Tampere University Hospital, PO Box 2000, Tampere, N33521 Finland; 2 Faculty of Medicine and Health Technology, Tampere University, Tampere, Finland; 3 Allergy Centre, Tampere University Hospital, Tampere, Finland

**Keywords:** Adult, Asthma, COPD, Guideline, Medication, Primary health care

## Abstract

**Background:**

Asthma and chronic obstructive pulmonary disease (COPD) are common diseases mostly treated in primary care. However, the usage patterns of drugs for obstructive airway diseases (R03 drugs) at the national level are not known.

**Objective:**

The aims of this study were to describe (1) for which diagnoses each class of R03 drugs were used, (2) the usage pattern of different drug classes for asthma and COPD, and (3) how often these medications were used without a diagnosis of asthma or COPD in Finland.

**Methods:**

We sent questionnaires that included questions on physician-diagnosed asthma and COPD to a random sample of 2000 Finnish subjects who had been dispensed R03 medications in the previous year. Details of R03 medications dispensed were retrieved from national registries.

**Results:**

Altogether, 803 subjects (40.6%) responded. Of these, 61.6% had asthma, 5.7% had both asthma and COPD, 5.1% had COPD, and 27.5% had neither asthma nor COPD. Among subjects with asthma or asthma and COPD, inhaled corticosteroids (ICS) were the most frequently dispensed class of drugs (93.7% and 97.8%, respectively). Even among subjects with COPD, ICS were dispensed as frequently (68.3%) as long-acting bronchodilators (70.7%). Antileukotrienes were dispensed mainly to asthmatic individuals only (18.4%) but far less frequently than ICS. The use of theophylline and roflumilast was rare.

**Conclusions:**

R03 medications are dispensed far more frequently for asthma than for COPD and often also for subjects without asthma or COPD. In line with guidelines, asthma is treated mainly with ICS, but there seems to be overuse of ICS for COPD.

**Supplementary Information:**

The online version contains supplementary material available at 10.1186/s12890-024-02870-9.

## Background

Obstructive airway diseases are among the most common chronic diseases worldwide. An estimated 300 million people worldwide have chronic obstructive pulmonary disease (COPD), and 262 million people have asthma [1]. In Finland, the prevalence of physician-diagnosed asthma and COPD was 11.2% and 2.6%, respectively, among adults in 2016 in a questionnaire study [2].

Much work has been done in Finland to improve asthma and COPD awareness, recognition and diagnostics through the National Asthma Programme (1994–2004), the National COPD Programme (1998–2007) and national guidelines [3, 4]. Among adults, both asthma and COPD are diagnosed and treated mainly in primary care and occupational health care. Physicians are always instructed to confirm asthma and COPD diagnoses with lung function tests [5, 6], and both spirometry and peak expiratory flow (PEF) monitoring are widely available in primary care [3]. Special reimbursement for asthma or COPD medications is dependent on diagnosis based on lung function measures. Thus, there is an economical incentive to always confirm the diagnosis with objective tests.

Group R03 drugs in the Anatomical Therapeutic Chemical (ATC) Classification System consist of drugs for obstructive airway diseases, including inhaled corticosteroids (ICS), long- and short-acting beta-agonists (LABA and SABA), long- and short-acting muscarinic antagonists (LAMA and SAMA), leukotriene receptor antagonists (LTRA), theophylline and roflumilast [7]. Most of these are indicated for both asthma and COPD, but their roles are different in these diseases. Treatment of asthma is based on maintenance treatment with ICS, and other classes are used as add-on maintenance drugs or as relievers [8]. Treatment of COPD is based on maintenance use of long-acting bronchodilators (e.g., LABA and LAMA), and ICS are used only if there is evidence of eosinophilic airway inflammation and recurrent acute exacerbations [9]. According to clinical experience, off-label use of R03 medications is quite common. There is little published data on how the use of these drugs is divided between diagnosed and undiagnosed adult patients, and off-label use is often related to the treatment of symptoms associated with airway infections [10–12]. Published data from paediatric populations also suggest that off-label use as short courses for airway infections is remarkable (19.2–38.1%) [13, 14].

The aims of this study were to describe (1) for which diagnoses each R03 drug class is used, (2) the usage pattern of different R03 drug classes for asthma and COPD, and (3) how often R03 medications are used without a diagnosis of asthma or COPD in Finland.

## Methods

### Study design and population

A postal questionnaire study was conducted in April 2017 [15]. The target group was a random sample of 2000 Finnish-speaking subjects aged 18–80 years to whom ATC group R03 medications were dispensed by pharmacies during the previous year and who resided in Finland. Reminders were sent twice. The exclusion criteria for the study were unsuccessful postal delivery of the questionnaire or nonanalysable data. The study protocol was approved by the Ethics Committee of Tampere University Hospital (approval number R15186). The sample size was originally determined based on another research question already published [15]. Based on a post-hoc power calculation the proportion of subjects reporting having asthma (67.4%) in this sample reflects the proportion in the population with an error margin of about 3% (95% CI from 64.0% to 70.6%).

### Postal questionnaire

The Finnish-language questionnaire was divided into four major sections: characteristics of the responder, different disease-specific questions, questions on medications used for treating obstructive airway diseases, and comorbidities. The English translation of the questionnaire has been previously published [15].

### Dispensing data

In Finland, all R03 medications are provided by pharmacies only with a prescription, and the dispenses with the identity of the subject are recorded by the Finnish Social Insurance Institution (FSII). The Finnish Social Insurance Institution (FSII) issues each person in Finland a unique social security number if they are permanent residents or entitled to it based on their employment status. The FSII collects registry on all prescription drug purchases made in the Finnish pharmacies. Each time a person buys a prescription medicine, the entitlement to reimbursements is checked. For each purchase, the personal identification data (i.e. the social security number) and the pharmaceutical information on the medicine are registered. The entitlement to the drug reimbursement is not related to persons socio-economic status or optional health insurances. The entitlement for special reimbursement of medical expenses is issued after the doctor has verified that the patient meets the predetermined disease specific criteria. For example, for asthma, patients may be issued entitlement for the special reimbursement of medical expenses if they have a doctor-verified asthma (based on typical symptoms and lung function testing), the patient has used anti-inflammatory medication for over six months and needs regular maintenance medication [16]. For both the responders and nonresponders, all dispensed medications in class R03 during 03/2016–02/2017 were requested from the FSII.

### Definitions

Age was defined as the age at the end of the year of the survey.

Smoking status was evaluated by two questions: “Have you ever smoked regularly?” and “Do you currently smoke?”. The responders were divided into never, former and current smokers.

Asthma was defined by a positive answer to the question “Do you have physician-diagnosed asthma?”.

COPD was defined by a positive answer to the question “Do you have a physician-diagnosed chronic obstructive pulmonary disease/COPD?”.

Other pulmonary diagnoses were assessed by the question “Do you have other physician-diagnosed pulmonary disease(s)? If yes, which?”.

In the statistical analysis, the responders were divided into four groups based on the pulmonary diagnoses they reported in the questionnaire (asthma, COPD, asthma and COPD, no obstructive airway disease).

### Statistical analysis

Statistical analyses were performed using SPSS Statistics version 26 (IBM Corp. Released 2019. IBM SPSS Statistics for Windows. Armonk, NY: IBM Corp). The Mann‒Whitney U test or Kruskal‒Wallis H test was used for continuous variables, and Pearson’s chi-square test or Fisher’s exact test was used for categorical variables (further post hoc analyses involved pairwise comparisons using the z test of two proportions with a Bonferroni correction or multiple Fisher’s exact tests (2 × 2) with a Bonferroni correction). A *p* value < 0.05 was considered statistically significant.

## Results

### Characteristics of responders and nonresponders

After excluding subjects due to death (*n* = 15) or unsuccessful postal delivery (*n* = 7), the corrected total sample size was 1978, and a total of 803 subjects responded to the questionnaire, yielding a response rate of 40.6%. The characteristics of the responders and nonresponders are shown in Table 1. Responders were slightly older and more often female than nonresponders. A higher proportion of responders had been dispensed ICSs, LTRAs and LABAs in comparison to nonresponders, but there were no differences in the dispensing of other drug classes between the groups.

**Table 1:** Characteristics of the responders and nonresponders

		**Responders**	**Nonresponders**	***p* value**
Total	N (% of the corrected total sample)	803 (40.6)	1175 (59.4)	n/a
Females	N (%)	492 (61.3)	642 (54.6)	0.003
Age^a^	Years	62 (50–70)	54 (41–66)	< 0.001
BMI^a^	kg/m^2^	27.3 (24.4–31.3)	n/a	n/a
Smoking status^b^	N (%)		n/a	n/a
Current		120 (14.9)		
Former		269 (33.5)		
Never		411 (51.2)		
Number of subjects dispensed different drug classes	N (%)			
ICS		662 (82.4)	854 (72.7)	< 0.001
LTRA		116 (14.4)	115 (9.8)	0.002
LABA		397 (49.4)	518 (44.1)	0.019
LAMA		78 (9.7)	119 (10.1)	0.763
SABA		513 (63.9)	771 (65.6)	0.428
SAMA		10 (1.2)	17 (1.4)	0.705
Theophylline		7(0.9)	14 (1.2)	0.496
Roflumilast		1(0.1)	3 (0.3)	0.525

*ICS* Inhaled corticosteroid, *LABA* Long-acting beta-agonist, *LAMA* Long-acting muscarinic antagonist, *LTRA *Leukotriene receptor antagonist, *OCS* Oral corticosteroid, *SABA* Short-acting beta-agonist, *SAMA* Short-acting muscarinic antagonist

^a^Median with IQR

^b^Subjects with complete answers regarding smoking habits

### Obstructive airway disease diagnoses among the users of R03 medications

The frequency of diagnoses of obstructive airway diseases and corresponding subject characteristics of the responders are presented in Table 2. Approximately two-thirds of the subjects reported having doctor-diagnosed asthma (61.6% reported asthma only and 5.1% reported both asthma and COPD), while only approximately 5% reported having COPD without asthma. Approximately one-quarter of the subjects reported no doctor-diagnosed obstructive airway disease. Seven (0.9%) responders reported having another physician-diagnosed pulmonary disease (one with pulmonary sarcoidosis, two with a history of lung transplantation, one with bronchiectasis, one with pleural plaques due to asbestosis, one with emphysema, and one with unspecified pulmonary fibrosis).

**Table 2:** Characteristics of the responders who were dispensed R03 medications according to their diagnoses

	**Asthma**	**Asthma and COPD**	**COPD**	**No obstructive airway disease**	**Overall ***p*** value**	**Post hoc *p* value < 0.05**
**Total**^**a**^	495(61.6)	46(5.7)	41(5.1)	221(27.5)	n/a	n/a
**Females**^**b**^	313(63.2)	20(43.5)	10(24.4)	149(67.4)	< 0.001	*p* < 0.05 for A and C C and No Dg A & C and No Dg
**Age**^c^ Years	61(49–70)	64(60–70)	69(65–76)	60(49–68)	< 0.001	*p* < 0.05 for C and No Dg A and C A & C and No Dg
**BMI**^c^ kg/m^2^	27.5 (24.4–31.2)	26.7 (23.8–30.7)	28.4 (22.9–31.9)	27.1(24.5–31.6)	0.741	
**Smoking status**^d,^^b^					< 0.001	n/a
** Current**	53 (10.7)	21 (45,7)	15 (36.6)	31 (14.4)		
** Former**	157 (31.7)	20 (43.5)	25 (61.0)	67 (31.0)		
** Never**	285 (57.6)	5 (10.9)	1 (2.4)	118 (54.6)		

Asthma (A), COPD (C), Asthma and COPD (A & C), no obstructive airway disease (No Dg)

^a^N (% of the corrected total sample)

^b^N (% within the DG Group)

^c^Median with IQR

^d^Subjects with complete answers regarding smoking habits

Subjects with COPD or both asthma and COPD were more often males, slightly older and, as expected, far less often never smokers in comparison to subjects with asthma or no obstructive airway disease.

### Diagnoses of obstructive airway diseases among subjects dispensed different classes of R03 medications

The total numbers of subjects who were dispensed different classes of R03 medications are presented in Table 1. The distribution of diagnoses of obstructive airway diseases among subjects dispensed each R03 drug class is presented in Table 3. Asthma was by far the most common diagnosis among the users of every drug class except LAMA, and at least three-quarters of users of ICS (76.9%), LTRA (81.8%) and LABA (76.1%) had doctor-diagnosed asthma or asthma and COPD. Among the subjects who were dispensed LAMA, COPD was as common as asthma, and the proportion of subjects with no obstructive airway diseases was clearly lower than for other drug classes. Almost a third of short-acting bronchodilator users (28.5% of SABA users and 30% of SAMA users) had no obstructive airway diseases.

**Table 3:** Diagnoses of obstructive airway diseases among subjects dispensed each R03 drug class (N (%))

	ICS	LTRA	LABA	LAMA	SABA	SAMA
Asthma (*n* = 495)	464 (70.1)	91 (78.4)	268 (67.5)	20 (27)	309 (60.2)	4 (40)
Asthma and COPD (*n* = 46)	45 (6.8)	4 (3.4)	34 (8.6)	27 (36.5)	34 (6.6)	1 (10)
COPD (*n* = 41)	28 (4.2)	1 (0.9)	29 (7.3)	21 (28.4)	24 (4.7)	2 (20)
No obstructive airway diseases (*n* = 221)	125 (18.9)	20 (17.2)	66 (16.6)	6 (8.1)	146 (28.5)	3 (30)

*ICS* inhaled corticosteroid, *LABA* long-acting beta-agonist, *LAMA* long-acting muscarinic antagonist, *LTRA* leukotriene receptor antagonist, *OCS* oral corticosteroid, *SABA* short-acting beta-agonist, *SAMA* short-acting muscarinic antagonist

### Dispensed R03 medications according to diagnosis

**Fig. 1:**
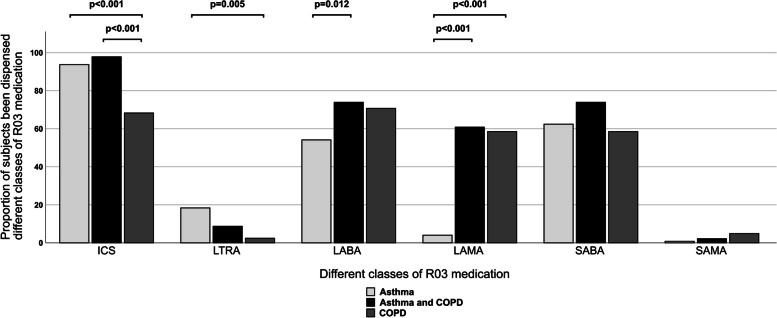
Proportions of subjects dispensed different classes of R03 medications according to obstructive airway disease diagnosis. (Note: only statistically significant pairwise comparisons are shown in Fig. 1. Post hoc analysis involves pairwise comparisons using Fisher’s exact tests (2 × 2) with a Bonferroni correction and statistical significance is accepted at *p* < 0.016667.)

Proportions of subjects dispensed different classes of R03 medications among subjects with asthma, asthma and COPD, and COPD are presented in Fig. 1. Exact numbers and data on subjects without a diagnosis of an obstructive airway disease are presented in Supplementary Table 1.

Among subjects with asthma, the most frequently dispensed class of medication was ICS (93.7%), followed by SABA (62.4%) and LABA (54.1%). The pattern of drug classes dispensed to subjects with both asthma and COPD was similar except for a much higher proportion of subjects who were also dispensed LAMA (60.9% for asthma and COPD vs. 4% for asthma, *p* < 0.001). In comparison, subjects with COPD only were less frequently dispensed ICS (68.3%), but among subjects with asthma and COPD, COPD patients were frequently dispensed both classes of long-acting bronchodilators (LABA and LAMA).

SABA were much more frequently dispensed in each diagnosis group in comparison to SAMA, and there was no difference between the diagnosis groups in terms of these drug classes. LTRA were dispensed most frequently among subjects with asthma (18.4%), but this was still far less common than the dispensing of ICS.

Among subjects with asthma, only 1% (*n* = 5) were dispensed LTRA without concomitant ICS. Only 5% (*n* = 27) of subjects with asthma or asthma and COPD were dispensed neither ICS nor LTRA. One subject was dispensed both SABA and LAMA, and the rest were dispensed SABA only. Among subjects with asthma or asthma and COPD, none were dispensed LABA only.

## Discussion

In this study, we found that approximately 60% of the subjects dispensed R03 medications in Finland reported having doctor-diagnosed asthma, 5% reported both asthma and COPD, and 5% reported COPD only. Approximately a quarter reported not having a doctor-diagnosed obstructive airway disease. In line with this, for all other R03 drug classes except LAMA, asthma was the most frequent indication. Among subjects with asthma or asthma and COPD, ICS were by far the most frequently dispensed class of drugs. Even among subjects with COPD, ICS were equally frequently dispensed as a long-acting bronchodilator. LTRA were dispensed mainly for asthmatic individuals only but far less frequently than ICS. The use of SAMA, theophylline and roflumilast was very infrequent.

Among the subjects who were dispensed R03 medications in this study, the proportion of patients with asthma (67.3%) was approximately six times higher than the proportion of patients with COPD (10.8%). This roughly six-to-one ratio was even higher than epidemiological data showing the prevalence of doctor-diagnosed asthma (11.2%) as approximately 4 times higher than that of COPD (2.6%) in surveys among Finnish adults [2]. One reason for the higher proportion of asthma patients in the current study in comparison to epidemiological data may be that a higher proportion of patients with mild COPD in comparison to mild asthma may not use any R03 medications and hence were not included in this study based on dispensed R03 medications.

One previous study from Portugal examined dispensed medications for respiratory diseases at the national level and estimated diagnoses based on treatment patterns [17]. In the study, the proportions of patients with likely any asthma (asthma only or asthma and COPD) and likely any COPD (COPD only or asthma and COPD) were approximately the same (51% vs. 54%, respectively). This one-to-one ratio between patients with asthma and COPD is much closer to the global estimate of asthma and COPD prevalence being approximately the same [1]. The difference between Finnish and global relative proportions of COPD and asthma is likely related to lower levels of smoking, better occupational hygiene, and higher rates of allergic diseases and asthma in Finland. In addition, local diagnostic practice favours the diagnosis of asthma in any subject with a significant bronchodilator response, although internationally, a fair proportion of subjects labelled as having COPD show significant reversibility [18]. In Finland, during the time of the study, it was easier to obtain special reimbursement for R03 medications if one had asthma in comparison to having COPD. Any asthma that had met the diagnostic criteria, patient had used anti-inflammatory maintenance medication for at least 6 months and needed regular maintenance medication, was granted the special reimbursement. On the contrary, in cases of COPD, the patient needed to have a fairly severe disease (FEV_1_ < 40% predicted, or FEV_1_ < 50% predicted and recurrent exacerbations despite of treatment) to get the special reimbursement. This discrepancy in the criteria may have directed physicians to make a diagnosis of asthma instead of COPD in cases with challenges in differential diagnostics or features of both diseases. The possible COPD related stigma may also have favoured the self-reporting of asthma over COPD.

Among patients with asthma or both asthma and COPD, ICS were the most frequently dispensed class of drugs. This is in line with national and international guidelines promoting the importance or maintenance of anti-inflammatory treatment [6, 8]. However, patients with COPD without asthma were dispensed ICS almost as frequently as LABA, which probably reflects previous guidelines in which ICS/LABA combinations were often recommended, and the use of dual bronchodilators was not as well established [19]. Thus, there is probably some degree of overuse of ICS in the treatment of COPD in Finland compared to what the current guidelines recommend [9].

Approximately 60% of patients with COPD or asthma and COPD were dispensed LAMA in line with current guidelines [9]. Although some LAMA-containing inhalers are also indicated for asthma, only 4% of patients with asthma were dispensed LAMA. This is a fairly low number, although tiotropium has been recommended for the treatment of asthma in the Finnish national guidelines since 2012 [20]. However, as the total number of subjects with asthma was much higher than the number of patients with COPD only, the number of LAMA users with only asthma was almost as high as the number of patients with only COPD.

Among the patients with asthma, SABA were dispensed to fewer patients than ICS, and none of the subjects used LABA without concurrent ICS. This is important since both overreliance on SABA and maintenance use of LABA without concurrent ICS are related to poor asthma outcomes [8, 21]. The use of SAMA was very infrequent for asthma, but approximately 5% of patients with COPD used SAMA. Overall, the role of anticholinergics has been advocated more in the treatment of COPD due to discussions on the role of increased cholinergic tone in COPD [22], but, actually, many asthmatic individuals also respond favourably to both short-acting and long-acting anticholinergics [23, 24]. The use of theophylline was also very infrequent and in line with current guidelines not recommending its use [8, 9]. Additionally, the use of roflumilast was very infrequent, although it is included in both national and international guidelines for the treatment of COPD [5, 9]. Its use is probably restricted by side effects and by the fact that a separate application for reimbursement of roflumilast is needed in Finland.

More than a quarter of the subjects who were dispensed R03 medications reported not having a diagnosis of asthma or COPD. Such use may include short courses of relievers or ICS for prolonged cough or airway infections, although there is no evidence to support this kind of prescription practice. Two previous studies, one from Australia [10] and one from the Netherlands [11], evaluated the prescribing of ICS to treat respiratory infections among subjects without chronic airway diseases. Both studies suggested that off-label use of ICS is common. In previous studies among children, acute airway infections have been a frequent off-label indication for R03 medications [13, 14]. In a Spanish study by Villamañán et al*.*, the off-label prescription of inhaled bronchodilators was common among hospitalised patients, and the most common indications were dyspnoea that was not related to asthma or COPD, respiratory infections, and heart failure [12]. In Finland, diagnostic assessment of suspected asthma in adults includes a two-week PEF monitoring with twice-daily measurements before and after SABA use [6]. Part of the reason for dispensed SABA to subjects without asthma or COPD may be related to investigations for possible asthma in which SABA are used to conduct the two-week PEF monitoring. A treatment trial with anti-inflammatory medication is used in asthma diagnostics [6, 8], so it is possible that part of the use of ICS among subjects not having reported asthma is explained by this.

A strength of this study is that it was based on a random sample of all subjects who were dispensed R03 medications in Finland. As there are no over-the-counter R03 medications available in Finland, all users of R03 medications were represented in the sample. The limitations of the study are that the overall response rate was approximately 40% (which is in line with that in other surveys these days), the diagnoses were self-reported and could not be verified from patient records and that the patients with milder COPD or asthma might be excluded from the study because of less frequent medicine purchases. Nonresponders were on average slightly younger and more often men. Nonresponders used anti-inflammatory medication and long-acting beta-2-agonists slightly less often, but there were no significant differences in the use of short-acting relievers or long-acting anticholinergics. We concluded that the responders represented the population of Finnish subjects using R03 medications, but there may have been a slight bias towards a more severe chronic airway disease among the responders and possibly a higher proportion of asthmatic individuals in comparison to COPD among responders when compared to nonresponders. Since special reimbursement for R03 medications in Finland is dependent on a firm diagnosis of asthma or COPD based on lung function measures, these diagnoses are generally reliable.

## Conclusions

Most of the subjects who were dispensed R03 medications reported being treated for asthma and far less frequently for COPD. Nevertheless, a quarter of subjects who were dispensed R03 medications reported not having a diagnosis of asthma or COPD. Among subjects reporting asthma, ICS were used most frequently, and guideline-based anti-inflammatory treatment was well adopted. Among subjects reporting COPD, the use of ICS was equally frequent as the use of long-acting bronchodilators, suggesting possible overuse of ICS.

### Supplementary Information

**Additional file 1: Supplementary Table 1.** Dispensing of R03 prescription medications for different pulmonary disease diagnoses.

## Data Availability

The datasets used and/or analysed during the current study available from the corresponding author on reasonable request.

## Abbreviations

ATC: Anatomical Therapeutic Chemical
CI: Confidence Interval
COPD: Chronic Obstructive Pulmonary Disease
FSII: Finnish Social Insurance Institution
ICS: Inhaled Corticosteroid
LABA: Long-Acting Beta-Agonist
LAMA: Long-Acting Muscarinic Antagonist
LTRA: Leukotriene Receptor Antagonist
OCS: Oral Corticosteroid
PEF: Peak Expiratory Flow
SABA: Short-Acting Beta-Agonist
SAMA: Short-Acting Muscarinic Antagonist
WHO: World Health Organization

## References

[1] GBD (2019). Diseases and Injuries Collaborators. Global burden of 369 diseases and injuries in 204 countries and territories, 1990–2019: a systematic analysis for the Global Burden of Disease Study 2019. Lancet Lond Engl 2020.

[2] Honkamäki J, Hisinger-Mölkänen H, Ilmarinen P, Piirilä P, Tuomisto LE, Andersén H (2019). Age- and gender-specific incidence of new asthma diagnosis from childhood to late adulthood. Respir Med.

[3] Haahtela T, Tuomisto LE, Pietinalho A, Klaukka T, Erhola M, Kaila M (2006). A 10 year asthma programme in Finland: major change for the better. Thorax.

[4] Kinnula VL, Vasankari T, Kontula E, Sovijarvi A, Saynajakangas O, Pietinalho A (2011). The 10-year COPD Programme in Finland: effects on quality of diagnosis, smoking, prevalence, hospital admissions and mortality. Prim Care Respir J J Gen Pract Airw Group.

[5] Working group appointed by the Finnish Medical Society Duodecim, the Finnish Respiratory Society. Chronic obstructive pulmonary disease (COPD). Current Care Guidelines. 2020. Available from: www.kaypahoito.fi Cited 2021.

[6] Working group appointed by the Finnish Medical Society Duodecim, the Finnish Respiratory Society, the Finnish Paediatric Society, the Finnish Society of Clinical Physiology. Asthma. Current Care Guidelines. 2022. Available from: www.kaypahoito.fi Cited 2022.

[7] WHO Collaborating Centre for Drug Statistics Methodology, Guidelines for ATC classification and DDD assignment 2023. Oslo, Norway, 2022. Available from: https://www.whocc.no/filearchive/publications/2023_guidelines_web.pdf.

[8] Global Initiative for Asthma (GINA). Global Strategy for Asthma Management and Prevention. www.ginasthma.org. 2022; Available from: www.ginasthma.org.

[9] Global Initiative for Chronic Obstructive Lung Disease. Global Strategy for Prevention, Diagnosis and Management of COPD. Glob Initiat Chronic Obstr Lung Dis GOLD. 2023 Available from: https://goldcopd.org/ Cited 2023.

[10] Poulos LM, Ampon RD, Marks GB, Reddel HK (2013). Inappropriate prescribing of inhaled corticosteroids: are they being prescribed for respiratory tract infections? A retrospective cohort study. Prim Care Respir J J Gen Pract Airw Group.

[11] Teichert M, Schermer T, van den Nieuwenhof L, De Smet PA, Wensing M (2014). Prevalence of inappropriate prescribing of inhaled corticosteroids for respiratory tract infections in the Netherlands: a retrospective cohort study. NPJ Prim Care Respir Med.

[12] Villamañán E, Sobrino C, Bilbao C, Fernández J, Herrero A, Calle M (2021). Off-label use of inhaled bronchodilators in hospitalised patients in Spain: a multicentre observational study. Eur J Hosp Pharm.

[13] Baiardi P, Ceci A, Felisi M, Cantarutti L, Girotto S, Sturkenboom M (2010). In-label and off-label use of respiratory drugs in the Italian paediatric population. Acta Paediatr.

[14] Schmiedl S, Fischer R, Ibáñez L, Fortuny J, Klungel OH, Reynolds R (2014). Utilisation and Off-Label Prescriptions of Respiratory Drugs in Children. PLoS ONE.

[15] Pakkasela J, Salmela P, Juntunen P, Karjalainen J, Lehtimäki L (2023). Adherence to treatment guidelines and good asthma control in Finland. Eur Clin Respir J.

[16] The Finnish Social Security Institute. Reimbursements for medicine expenses. 2023. Available from: https://www.kela.fi/medicine-expenses Cited 2023.

[17] Sá-Sousa A, Amaral R, Almeida R, Freitas A, Almeida FJ (2022). Prescribing patterns of medication for respiratory diseases: cluster analysis of the Portuguese electronic prescription database. Eur Ann Allergy Clin Immunol.

[18] Tashkin DP, Celli B, Decramer M, Liu D, Burkhart D, Cassino C (2008). Bronchodilator responsiveness in patients with COPD. Eur Respir J.

[19] Working group appointed by the Finnish Medical Society Duodecim, the Finnish Respiratory Society. Chronic obstructive pulmonary disease (COPD). Current Care Guidelines. 2014 Available from: www.kaypahoito.fi Cited 2021.

[20] Working group appointed by the Finnish Medical Society Duodecim, the Finnish Respiratory Society, the Finnish Paediatric Society, the Finnish Society of Clinical Physiology. Asthma. Current Care Guidelines. 2013. Available from: www.kaypahoito.fiCited 2021.

[21] Janson C, Menzies-Gow A, Nan C, Nuevo J, Papi A, Quint JK (2020). SABINA: An Overview of Short-Acting β2-Agonist Use in Asthma in European Countries. Adv Ther.

[22] Gross NJ, Co E, Skorodin MS (1989). Cholinergic Bronchomotor Tone in COPD. Chest.

[23] Kirkland SW, Vandenberghe C, Voaklander B, Nikel T, Campbell S, Rowe BH (2017). Combined inhaled beta‐agonist and anticholinergic agents for emergency management in adults with asthma. Cochrane Database Syst Rev.

[24] Oba Y, Anwer S, Maduke T, Patel T, Dias S (2022). Effectiveness and tolerability of dual and triple combination inhaler therapies compared with each other and varying doses of inhaled corticosteroids in adolescents and adults with asthma: a systematic review and network meta-analysis. Cochrane Database Syst Rev.

