# Supplementary material for: Diagnoses and prescription patterns among users of medications for obstructive airway diseases in Finland
Source: BMC Pulm Med. 2024 Jan 31;24:64. doi: 10.1186/s12890-024-02870-9 (PMC10829254; doi:10.1186/s12890-024-02870-9)
Supplement: Supplementary file 1 — Additional file 1: Supplementary Table 1. Dispensing of R03 prescription medications for different pulmonary disease diagnoses. [file 12890_2024_2870_MOESM1_ESM.docx]

# Supplementary material for submission ID 0f3051af-aa4e-49a4-bc33-914ef87e6eaf

**Manuscript title:**

Diagnoses and prescription patterns among users of medications for obstructive airway diseases in Finland

**Authors:**

Pekka Juntunen, MD1,2*, Petri Salmela, MD1,2*, Johanna Pakkasela, MD1,2, Jussi Karjalainen MD, PhD2,3, Lauri Lehtimäki MD, PhD2,3

1 Department of Respiratory Medicine, Tampere University Hospital, Tampere, Finland.

2 Faculty of Medicine and Health Technology, Tampere University, Tampere, Finland.

3 Allergy Centre, Tampere University Hospital, Tampere, Finland.

*Shared first authorship.

Corresponding author: Dr Pekka Juntunen, MD
 Department of Respiratory Medicine
 Tampere University Hospital
 PO Box 2000
 FIN-33521 Tampere, FINLAND
 E-mail: [pjuntunen@fimnet.fi](mailto:pjuntunen@fimnet.fi)

**Supplementary Table 1.** Dispensing of R03 prescription medications for different pulmonary disease diagnoses.

| **Pulmonary diagnosis** | **Drug class** | **Total***  **(N=803)** |
| --- | --- | --- |
|  |  |  |
| Asthma  (N=495) | ICS | 464 (93.7) |
|  | LTRA | 91 (18.4) |
|  | LABA | 268 (54.1) |
|  | LAMA | 20 (4.0) |
|  | SABA | 309 (62.4) |
|  | SAMA | 4 (0.8) |
|  |  |  |
| Asthma and COPD (N=46) | ICS | 45 (97.8) |
|  | LTRA | 4 (8.7) |
|  | LABA | 34 (73.9) |
|  | LAMA | 28 (60.9) |
|  | SABA | 34 (73.9) |
|  | SAMA | 1 (2.2) |
|  |  |  |
| COPD  (N=41) | ICS | 28 (68.3) |
|  | LTRA | 1 (2.4) |
|  | LABA | 29 (70.7) |
|  | LAMA | 24 (58.5) |
|  | SABA | 24 (58.5) |
|  | SAMA | 2 (4.9) |
|  |  |  |
| No obstructive airway diseases  (N=221) | ICS | 125 (56.6) |
|  | LTRA | 20 (9.0) |
|  | LABA | 66 (29.9) |
|  | LAMA | 6 (2.7) |
|  | SABA | 146 (66.1) |
|  | SAMA | 3 (1.4) |

* N (% within diagnosis group)

Abbreviations used in Supplementary Table 1: ICS, inhaled corticosteroid; LABA, long-acting beta-agonist; LAMA, long-acting muscarinic antagonist; LTRA, leukotriene receptor antagonist; OCS, oral corticosteroid; SABA, short-acting beta-agonist; SAMA, short-acting muscarinic antagonist.
